# A Bioactivity-Based Method for Screening, Identification of Lipase Inhibitors, and Clarifying the Effects of Processing Time on Lipase Inhibitory Activity of Polygonum Multiflorum

**DOI:** 10.1155/2016/5965067

**Published:** 2016-01-26

**Authors:** Yan-xu Chang, Ai-hua Ge, Yan Jiang, John Teye Azietaku, Jin Li, Xiu-mei Gao

**Affiliations:** ^1^Tianjin State Key Laboratory of Modern Chinese Medicine, Tianjin University of Traditional Chinese Medicine, Tianjin 300193, China; ^2^Tianjin Key Laboratory of Phytochemistry and Pharmaceutical Analysis, Tianjin University of Traditional Chinese Medicine, Tianjin 300193, China

## Abstract

Traditional Chinese medicine (TCM) has been used for the treatment of many complex diseases. However, the bioactive components are always undefined. In this study, a bioactivity-based method was developed and validated to screen lipase inhibitors and evaluate the effects of processing on the lipase inhibitory activity of TCM by ultrahigh performance liquid chromatography coupled with quadrupole-time-of-flight mass spectrometry and fraction collector (UHPLC/Q-TOF-MS-FC). The results showed that both* Polygonum multiflorum* and processed* P. multiflorum* extracts had inhibitory effect against lipase with IC_50_ values of 38.84 *μ*g/mL and 190.6 *μ*g/mL, respectively. Stilbenes, phenolic acid, flavonoids, and anthraquinones were considered to be the potential lipase inhibitors. Eleven potential lipase inhibitors were simultaneously determined by UHPLC. Principal component analysis (PCA) was employed in exploring the effects of processing time on lipase inhibitory activity of* P. multiflorum*. Compared with conventional methods, a bioactivity-based method could quantitatively analyze lipase inhibitory activity of individual constituent and provide the total lipase inhibitory activity of the samples. The results demonstrated that the activity integrated UHPLC/Q-TOF-MS-FC method was an effective and powerful tool for screening and identifying lipase inhibitors from traditional Chinese medicines.

## 1. Introduction

Nowadays, obesity presents as a main health problem in the world, which is often associated with pathological disorders [1, 2]. Obesity is caused by excessive energy intake over low energy expenditure, leading to the accumulation of fat in the body. The fat contained in food is the excess energy source. In order to reduce fat accumulation in the body, the digestion and absorption of the fat after food intake should be prevented [3]. At present, lipase has been selected as a therapeutic target for the prevention of fat digestion. There are some synthetic pancreatic lipase inhibitors such as orlistat, used widely as effective antiobesity drugs [3, 4]. However, long term usage of these drugs has various side effects such as liver toxicity, abdominal distention, and borborygmus [5]. From this perspective, it is necessary to screen green and safe lipase inhibitors from natural products.

For this purpose, many plants including traditional Chinese medicines (TCMs) have been used for the treatment of obesity [6–9].* Polygonum multiflorum* (Heshouwu in Chinese), obtained from the root of* P. multiflorum* Thunb., is an example of a TCM used over centuries for the treatment of various kinds of diseases in China [10]. The main components in* P. multiflorum* are stilbenes, phenolic acids, flavonoids, and anthraquinones [11–13]. Phenolic compounds, such as gallic acid and catechin, have antioxidant activity* in vivo* in previous reports [14, 15]. Moreover, anthraquinones have anti-inflammatory, hemostatic, laxative, and antibacterial activities [16]. Specifically, stilbenes are known for their effect in treating neurodegenerative diseases, such as Alzheimer's disease and Parkinson's disease [17–19]. They are the active components contributing to the pharmacological effects of* P. multiflorum*. There are reports on the lipid-lowering activity of* P. multiflorum* for the treatment of hyperlipidemia in animal and cell experiments [20–22]. However, the lipid regulation mechanisms were still not clearly elucidated. Therefore, lipase was selected as a key enzyme to screen lipase inhibitors for elucidating the lipid regulation mechanisms of* P. multiflorum*.

It is a great challenge for separating, screening, and identifying lipase inhibitors from TCMs due to the complexity and variability of components. Besides, traditional screening method of isolating, purifying, and evaluating lipase inhibitors using animal and cell models is time-consuming and labor-intensive and this makes it difficult screening lipase inhibitors in TCMs directly [23]. The lipase inhibitors may be lost during the process of isolation and purification. Thus, it is very necessary to establish a rapid and simple method in obtaining relatively pure compounds from bioassays. It could be applied directly to screen lipase inhibitors from TCMs. In order to overcome the disadvantages flogged with the use of conventional methods, HPLC coupled with bioassays was used to screen bioactive components [24, 25]. With the development of modern techniques, UHPLC-Q-TOF/MS has been widely used in the screening of components from TCMs not only for its high peak capacity, high sensitivity, and resolution but also for the exact mass determination by the Q-TOF/MS [26, 27]. Furthermore, the fraction collector (FC) is a useful tool for the rapid sample preparation to test the activity of chromatographic fractions.

In this study, bioactivity-based UHPLC-Q-TOF/MS-FC method was applied to screen the lipase inhibitors and evaluate the effects of processing time on inhibitory lipase activity of* P. multiflorum*. In the approach, UHPLC coupled with FC was used for the rapid sample preparation of the bioassays. UHPLC-Q-TOF/MS was employed to provide chemical information on the lipase inhibitors from TCMs. Finally, the screened and identified lipase inhibitors were simultaneously determined by UHPLC-PDA to evaluate the effects of processing on the quality of* P. multiflorum*. The method could reflect the bioactivity of the whole extract and individual components, structure-activity relationships, concentration-effect relationships, and the concentration of individual compounds. This novel technique may help to discover lipase inhibitors rapidly and efficiently and help evaluate the effects of processing on the quality of TCMs and its benefits in drug research and development.

## 2. Experimental

### 2.1. Plant Materials

*P. multiflorum* was purchased from Anguo TCM market (Hebei, China) and authenticated by Professor Lin Ma (Tianjin University of Traditional Chinese Medicine).* P. multiflorum* was processed by black soybean decoction according to the Chinese Pharmacopoeia, into the processed* P. multiflorum*. The procedure was as follows:* P. multiflorum* was mixed with black bean extract for 24 h (10 g black bean extracted with 200 mL water twice), it was finally steamed for 36 h, and then the processed* P. multiflorum* was obtained. 5.0 kg processed* P. multiflorum* and* P. multiflorum* powder were fluxed with 5 L 95% ethanol and refluxed with 8 L 60% ethanol for 2 h, respectively. Then, the extraction was combined, condensed, and lyophilized. The extraction yield was 17.2% for* P. multiflorum* and 9.45% for the processed* P. multiflorum*. Finally, the plant materials were obtained.

### 2.2. Chemicals and Reagents

Acetonitrile, purchased from Merck (Darmstadt, Germany), and methanol purchased from (Tianjin Concord Science Co. Ltd., Tianjin, China) were of HPLC grade. Formic acid of chromatographic grade was purchased from Tedia Company Inc. (Tedia, Fairfield, OH, USA). Deionized water was purified with a Milli-Q Academic ultrapure water system (Millipore, Milford, MA, USA). Reference standards of gallic acid, catechin, epicatechin, polydatin, 2,3,5,4′-tetrahydroxystilbene-2-O-*β*-D-glucoside, resveratrol, emodin-8-O-glucoside, physcion-8-O-glucoside, rhein, emodin, and physcion (purity > 98%) were purchased from Chengdu Must Biotechnology Co., Ltd. (Chengdu, China). Lipase (type II, L3126) was purchased from Solar Bio Science Co. Ltd. (Beijing, China). Orlistat (purity > 98%) was obtained from Sigma-Aldrich (St. Louis, Missouri, USA). Other reagents were of analytical grade and obtained commercially.

### 2.3. Sample Preparations

#### 2.3.1. Preparation of the* P. multiflorum* Extract

The extracts of processed* P. multiflorum* and* P. multiflorum* (0.05 g) were weighed accurately and extracted with 10 mL 70% (v/v) methanol ultrasonically for 40 min. After centrifugation at 14,000 rpm for 10 min, the supernatants were filtered through 0.22 *μ*m filter. Then, the solutions were injected into the UHPLC system for the fraction collection and the quality control of* P. multiflorum* or diluted to obtain the suitable concentrations for bioassays. The contents of gallic acid, catechin, epicatechin, polydatin, 2,3,5,4′-tetrahydroxystilbene-2-O-*β*-D-glucoside, resveratrol, emodin-8-O-glucoside, physcion-8-O-glucoside, rhein, emodin, and physcion in* P. multiflorum* extract were 0.146 mg/g, 0.035 mg/g, 0.140 mg/g, 0.138 mg/g, 57.497 mg/g, 0.192 mg/g, 7.885 mg/g, 0.584 mg/g, 0.052 mg/g, 0.444 mg/g, and 0.656 mg/g, respectively. Those in processed* P. multiflorum* extract were 0.528 mg/g, 0.001 mg/g, 0.03 mg/g 7, 0.076 mg/g, 29.824 mg/g, 3.352 mg/g, 0.215 mg/g, 0.054 mg/g, 1.354 mg/g, and 2.074 mg/g, respectively.

#### 2.3.2. Preparation of the Fractions

When the* P. multiflorum* extract was injected into the UHPLC system, the fraction collector (BSZ-100, Shanghai QingpuHuxi Instrument, Shanghai, China) was used for the fraction collection by setting the time interval at 20 s. Then, the fractions were collected and evaporated to dryness by nitrogen gas. The residues were reconstituted and diluted for bioassays.

#### 2.3.3. Preparation of Substrate and Enzyme Solutions

4-Methylumbelliferyl oleate (4.406 mg) was accurately weighed and dissolved by Tris-HCl solution (pH 8.0, 1.3 mM NaCl, and 1.3 mM CaCl_2_) with the final concentration of 0.1 mM. 100 mg lipase was dissolved with deionized water and the insoluble substances were removed by centrifugation at 14,000 rpm for 10 min. Finally, the concentration of enzyme solution was 1.0 mg/mL.

#### 2.3.4. Preparation of Standard Solutions

Gallic acid, catechin, epicatechin, polydatin, 2,3,5,4′-tetrahydroxystilbene-2-O-*β*-D-glucoside, resveratrol, emodin-8-O-glucoside, physcion-8-O-glucoside, rhein, emodin, and physcion with the concentration of 1.0 mg/mL were prepared in methanol for quality control. Rhein, emodin, and physcion were dissolved in DMSO (Dimethyl Sulfoxide) with suitable concentrations. The reference standards solution was diluted serially with 10% methanol for the bioassays. The concentrations of DMSO in a series of samples at different concentrations were less than 0.1% (v/v). Orlistat was used as the positive control of lipase, respectively. It was prepared with 10% methanol and diluted to a series of different concentrations.

### 2.4. UHPLC Analysis

The separation of* P. multiflorum* extract was operated on a Waters Acquity UHPLC System (Waters Co., Milford, MA). UHPLC system was equipped with PDA detector of 190–400 nm. An Acquity UHPLC BEH C18 (1.7 *μ*m, 2.1 × 50 mm) column was employed for the separations. The mobile phases were made up of 0.1% (v/v) formic acid aqueous solution (A) and acetonitrile (B) with a gradient elution of 0-1 min, 5%-5% B; 1–7 min, 5%–20% B; 7–10 min, 20%–35% B; 10–15 min, 35%–65% B; 15–18 min, 65%–80% B; and 18–18.1 min, 80%–5% B. Reequilibration time after gradient elution was 5 min. The flow rate was 0.3 mL min^−1^. The column temperature was set at 30°C. The injection volume was 1 *μ*L.

### 2.5. UHPLC-Q-TOF-MS Analysis

The components in* P. multiflorum* extract were identified by an Agilent Q-TOF-MS system. Aglient 6520 Q-TOF mass spectrometer (Agilent Corporation, Santa Clara, CA, USA) coupled with the Agilent 1290 HPLC via an ESI interface was used to obtain chemical information. The mobile phases, flow rate, column temperature, and injection volume were the same as in the UHPLC analysis. The detection wavelengths were set at 254 for emodin and physcion and at 280 nm for other components. The gradient elution was set as follows: 0–4 min, 3%–12% B; 4–8 min, 12%–15% B; 8–13 min, 15%–25% B; 13–16 min, 25%–50% B; and 16–20 min, 50%–80% B. Reequilibration time after gradient elution was 5 min. The ESI-MS spectra were obtained in both positive and negative ion modes to provide complete information for the compounds identification. The Q-TOF-MS analysis conditions were set as follows: capillary voltage, 4500 V; fragmentor voltage, 175 V; skimmer voltage, 65 V; drying gas temperature, 350°C; drying gas (N_2_) flow rate, 10 L/min; nebulizer gas pressure, 35 psig; and octopole RF, 750 V. The mass range was* m/z* 100–1000. The ions [M-H]^−^ were selected as precursor ions and subjected to target-MS/MS analysis.

### 2.6. Method Validation

The method validation including linearity, limits of detection (LOD), limits of quantification (LOQ), repeatability, precision, stability, and recovery was performed on the basis of US Pharmacopeia recommendations and guidelines.

#### 2.6.1. Linearity, Repeatability, LODs, and LOQs

The calibration curves were constructed with the diluted concentrations of the reference compounds by plotting the peak areas (*y*) versus the corresponding concentration (*x*, *μ*g/mL). The repeatability was evaluated by six independent sample solutions and expressed as the relative standard deviation (RSD). The stocks solution of the reference compounds was diluted to a certain concentration at the signal-to-noise (*S*/*N*) ratio of 3 and 10, respectively. Six independent sample solutions were evaluated for the repeatability test.

#### 2.6.2. Precision, Stability, and Recovery

The intraday and interday variability were used for the evaluation of the precision. Intraday and interday precision were assessed by the standard solutions of three different concentrations (low, medium, and high concentration) within one day and over three consecutive days, respectively. The variability was expressed as RSD. The stability experiment was determined for the standard solutions at three different concentrations at the time interval of 0, 2, 4, 6, 8, 12, and 24 h at room temperature. The recovery was determined by spiking three different concentrations (80%, 100%, and 120%) of the eleven compounds into certain amount (0.05 g) of the* P. multiflorum* and then the mixed solutions were extracted and analyzed by the above method. Finally, the recovery was calculated by the formula: recovery (%) = (found amount – original amount)/spiked amount × 100%.

#### 2.6.3. Repeatability and Recovery of Fraction Collections

The* P. multiflorum* extract was injected into the UHPLC system and then collected by the FC. Six batches (six times of the collection were used as a batch) were collected to evaluate the repeatability of the fraction collection method. Eleven known components were selected as markers to determine the yield of the collected fractions. The same peak was divided into several fractions, which were combined and condensed to dryness by nitrogen gas. The recovery of the fraction was assessed by the ratio of the peak content in the reconstituted fraction and in the* P. multiflorum* extract, which was employed to evaluate the loss in the process of fraction collection.

#### 2.6.4. Sample Analysis

The* P. multiflorum* extract and processed* P. multiflorum* extract (with different processing times) were analyzed by the developed method. The concentrations of the eleven components were determined.

#### 2.6.5. Assay for Lipase Inhibitory Activity

The assay of lipase was performed according to previous method with slight modifications [28]. 25 *μ*L test samples and 25 *μ*L lipase solution were mixed together. Then, 50 *μ*L 4-methylumbelliferyl oleate was added to the mixture. After incubating at 25°C for 20 min, 100 *μ*L sodium citrate (0.1 M, pH 4.2) was added to stop the reaction. Finally, the amount of 4-methylumbelliferone released by lipase was measured by microplate reader at an excitation wavelength of 355 nm and an emission wavelength of 460 nm. The inhibitory activity was calculated by the formula: inhibition (%) = [1 − (*A*_sample_/*A*_control_)] × 100, where *A*_sample_ and *A*_control_ were absorbance of the test sample and the control, respectively. Orlistat was used as the positive control.

### 2.7. Statistical Analysis

In the bioassay experiments, all the analysis was carried out in triplicate. All values were expressed in the form of mean ± standard deviation (SD); IC_50_ values were determined by Graph Pad Prism 5 software.

## 3. Results and Discussion

### 3.1. Method Validation

#### 3.1.1. Linearity, LODs, and LOQs

The information of linearity, LODs, and LOQs was listed in Table 1. All compounds had a good linear relationship within the linear range with *R* ^2^ > 0.9955. The LODs and LOQs of the compounds were in the range of 0.06 to 1.85 *μ*g/mL and 0.22 to 5.56 *μ*g/mL, respectively.

#### 3.1.2. Precision, Stability, and Recovery

As shown in Table 2, all the RSD values of intraday and interday precision were less than 5% and 4.91%, respectively. The intraday and interday accuracies were in the range of 94.2%–106% and 93.7%–107%. The results indicated that the method was precise for the quantitative analysis and fraction collection of the* P. multiflorum* extract. The RSDs of the stability of the analytes were less than 4.98% and the accuracy was in the range from 93.7% to 106%, demonstrating that the sample solutions were stable within 24 h at room temperature. The RSD values of the recoveries at three different concentrations were less than 4.49% and the recoveries were in the range of 95.3% to 105% (Table 3), which illustrated that the extraction method was of high accuracy.

#### 3.1.3. Recovery of Fraction Collections

The results of the repeatability of enriched fractions were no more than 3.97% (Table 1), demonstrating that the fraction collection method was reproducible for the rapid sample preparation. The recoveries of the 11 fractions were higher than 71.4%, illustrating that the loss in the enriching process was acceptable and the process was reproducible (data was not given).

### 3.2. Lipase Inhibitory Activity of* P. multiflorum* Extract

The* lipase inhibitory activity* of* P. multiflorum* and processed* P. multiflorum* extracts at the concentration of 4–5000 *μ*g/mL was investigated. The results showed that both* P. multiflorum* and processed* P. multiflorum* extracts have the inhibitory effect against lipase. IC_50_ values of* P. multiflorum* and processed* P. multiflorum* extracts were 38.84 *μ*g/mL and 190.6 *μ*g/mL, respectively. These results indicated that activity of* P. multiflorum* was better than processed* P. multiflorum*. The contents of 11 typical constituents were determined by UPLC-PDA. It was found that the contents of catechin, epicatechin, polydatin, 2,3,5,4′-tetrahydroxystilbene-2-O-*β*-D-glucoside, resveratrol, physcion-8-O-glucoside, and emodin-8-O-glucoside in* P. multiflorum* were higher than those in processed* P. multiflorum*. The contents of gallic acid, rhein, emodin, and physcion in* P. multiflorum* were lower than those in processed* P. multiflorum*. It was concluded that* P. multiflorum* extract showing better activity than processed* P. multiflorum* is related to contents of some lipase inhibitors. Thus, it was necessary to screen potential lipase inhibitors and* P. multiflorum* extract was selected for the bioactive fraction collection by the UHPLC-FC.

### 3.3. Lipase Inhibitory Activity

**Figure 1:**
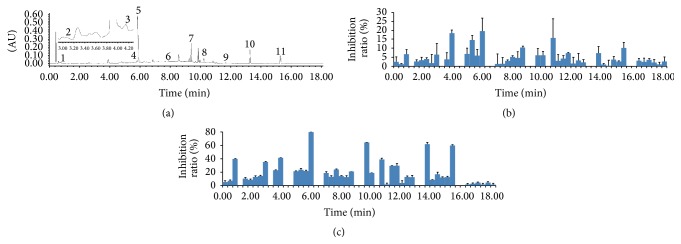
The chromatogram (a) and lipase inhibitory effect of enriched fractions for 40 times (b) and 60 times (c).

The extract was injected in UHPLC for the separation of the components and all fractions were collected for the bioassays. The lipase inhibitory effect was expressed as inhibition ratio (%), which could be calculated by the above mentioned formula. The time interval of fraction collector was set at 20 s according to the resolution and peak shape in the chromatogram. Thus, the relative purity of the fractions was guaranteed for the bioassays. The fractions from the same peak were combined. From Figure 1, we could observe that the lipase inhibitory activity of the fractions samples that were enriched for 40 and 60 times showed a nonlinear dose-dependent relationship. That is, with the enriched times increasing, the bioactivity of some fractions increased while some did not. When the* P. multiflorum* extract was enriched for 60 times, the lipase inhibitory activity of some fractions was higher than 50%. As can be seen from Figure 1, the fractions of peaks 5, 6, 7, 8, 9, 10, and 11 showed strong inhibition of lipase, while the peaks 1, 2, 3, and 4 showed relatively weak inhibitory effect of lipase. These fractions containing 20 bioactive components were screened directly and might be considered as the antilipase active components in* P. multiflorum* extract. As a result, the established method could be used to screen potential lipase inhibitory TCMs and bioactive components.

### 3.4. Identification of the Bioactive Components

The identification of the components in* P. multiflorum* extract was carried out by UHPLC-Q-TOF-MS. The 70% methanol extract of* P. multiflorum* was employed to obtain the total ion chromatogram (TIC) of MS study and MS/MS study of the fragment ion. As can be seen from Table 4, 11 compounds were identified or characterized tentatively according to previous reports. Among the identified compounds, there were 1 phenolic acid, 2 flavonoids, 3 stilbenes, and 5 anthraquinones.

Peaks 1, 2, and 3 were unambiguously identified as gallic acid, catechin, and epicatechin, respectively, by comparing mass spectrometric behavior and retention time with the reference compounds. Peak 4 produced [M-H]^−^ at* m/z* 389.1168 with the molecular formula C_20_H_21_O_8_. A loss of C_6_H_10_O_5_ in MS/MS study was confirmed to be a hexose neutral loss. Peak 4 was tentatively identified as polydatin by comparing the MS data with literature [29]. The MS and MS/MS spectra of peak 5 gave diagnostic ions at* m/z* 405.1 and 243.1, of which the elemental compositions were C_20_H_21_O_10_ and C_14_H_11_O_4_. Further fragmentation produced characteristic ions at* m/z* 215 and 137. Peak 5 was tentatively characterized as 2,3,5,4′-tetrahydroxystilbene-2-O-*β*-D-glucoside [30]. The negative MS spectra of peak 6 exhibited a parent ion [M-H]^−^ at* m/z* 227.2, while a fragment ion at* m/z* 185.2, 143.1, and 119.2 was observed in MS/MS spectra. Based on the previous literature, peak 6 was identified as resveratrol [31]. Emodin-8-O-glucoside (peak 7) and physcion-8-O-glucoside (peak 8) were identified certainly by comparing the characteristic mass spectra with the reference standards. The negative MS spectra of peak 9 showed a parent ion [M-H]^−^ at* m/z* 283.1 and fragment ion at* m/z* 239.0 was observed in MS/MS spectra, which was tentatively characterized as rhein according to the previous report [32]. The characteristic fragment ions of 283.1, 269.0, and 240.0, the elemental composition, and characteristic fragmentation behaviors could be employed to identify the anthraquinone derivatives. Two components, emodin (peak 10) and physcion (peak 11), were unambiguously identified by comparing mass spectra and retention times with their reference compounds.

As a result, peaks of 1–11 were identified as gallic acid, catechin, epicatechin, polydatin, 2,3,5,4′-tetrahydroxystilbene-2-O-*β*-D-glucoside, resveratrol, emodin-8-O-glucoside, physcion-8-O-glucoside, rhein, emodin, and physcion, respectively. As a more sensitive and reliable method, the integrated MS identification and activity screening method could be used in screening bioactive components and identification of components from natural products. The chemical information of* P. multiflorum* extract also provided helpful information of the activity study and could be used in the compound identification of other similar TCMs.

### 3.5. The Contribution of Lipase Inhibitory Activity

The inhibitory effect of positive control orlistat against lipase was determined and IC_50_ value was calculated by the software Graph Pad Prism 5. The IC_50_ value of orlistat was 0.167 *μ*g/mL. The IC_50_ of orlistat was defined as a potency unit. The potency of other concentrations of orlistat was calculated by the potency unit. The potency calibration curve was constructed by plotting the potency (*y*) against the corresponding inhibition ratio (*x*). The calibration curve of orlistat was *y* = 0.0033*x* ^3^ − 0.5271*x* ^2^ + 28.111*x* − 497.27 (*R* ^2^ = 0.9956). Then, the potency of the enriched fractions was determined by the curve mentioned above. Therefore, the relative percentages of inhibition of* lipase of* all identified fractions expressed potency unit of orlistat equivalent* antilipase* capacity according to standard potency curves. The contribution of individual fractions was calculated by the formula: contribution (%) = potency of individual fraction/the total lipase inhibitory × 100%. The results showed that the 2,3,5,4′-tetrahydroxystilbene-2-O-*β*-D-glucoside played the most important role in the lipase inhibitory effect contributing more than 70% while phenolic acid, flavonoids, and other two stilbenes made relatively little contribution to the whole activity. Anthraquinones like emodin-8-O-glucoside, emodin, and physcion made moderate contribution to the total activity contributing less than 10% of the total activity. The possible reasons might be that the 2,3,5,4′-tetrahydroxystilbene-2-O-*β*-D-glucoside does have strong inhibitory effect against lipase and the content of it was the highest among all the components. As a result, 2,3,5,4′-tetrahydroxystilbene-2-O-*β*-D-glucoside and anthraquinones were the main components exhibiting lipase inhibitory effect. The results were consistent with that of the activity of the fractions.

### 3.6. Confirmation of the Bioactivity of the Bioactive Components

In order to validate the results obtained from the established method, the bioassays of the reference standards of the bioactive components (purity > 98%) from the* P. multiflorum* extract were carried out at the same time. Eight different diluted concentrations of the bioactive components of the lipase inhibitory effect were assessed. The effective concentration at which lipase was inhibited by 50% was defined as IC_50_ values of the components against lipase. The inhibitory effect of the compounds was expressed as inhibition ratio (%). The results were listed in Table 4. Except gallic acid, catechin, and epicatechin, the lipase inhibition ratio of the compounds was above 50% at the concentration of 1.0 mg/mL, which was consistent with the inhibitory activity of the bioactive fractions. The IC_50_ values of the bioactive components with the lipase inhibition ratio more than 50% were calculated. The lipase inhibitory effects of rhein, emodin-8-O-glucoside, emodin, 2,3,5,4′-tetrahydroxystilbene-2-O-*β*-D-glucoside, and physcion-8-O-glucoside were stronger with their IC_50_ values being 231.0, 296.0, 349.7, 350.5, and 421.5 *μ*g/mL, respectively. Those of polydatin, resveratrol, and physcion were 1318, 564.3, and 812.8 *μ*g/mL, of which their inhibitory effect against lipase was relatively weaker. As a result, stilbenes and anthraquinones were screened to have the potential inhibitory effect against lipase from the* P. multiflorum* extract. The results might be of significant value to clinical drug use.

### 3.7. The Effects of Processing on Quality of* P. multiflorum* Extract

**Figure 2:**
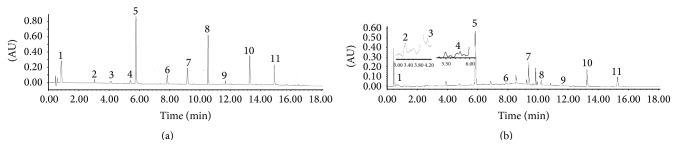
The UHPLC chromatogram of the quantitative analysis of 11 components. (a) The mixed standard solutions of 11 components. (b) The* P. multiflorum* extract (1 = gallic acid, 2 = catechin, 3 = epicatechin, 4 = polydatin, 5 = 2,3,5,4′-tetrahydroxystilbene-2-O-*β*-D-glucoside, 6 = resveratrol, 7 = emodin-8-O-glucoside, 8 = physcion-8-O-glucoside, 9 = rhein, 10 = emodin, and 11 = physcion).

**Figure 3:**
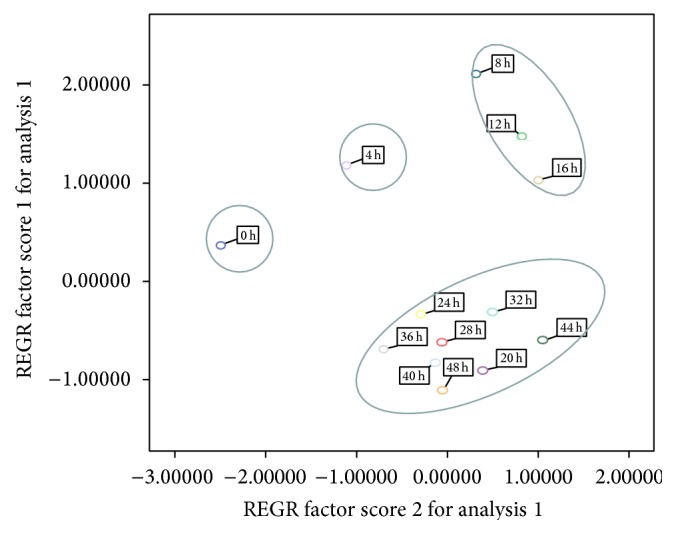
The score plot of* P. multiflorum* with different processing times.

11 components were screened and identified by the developed UHPLC-Q-TOF/MS-FC method (Figure 2). In order to clarify the effect of processing on lipase activity, the different processing times on contents of lipase inhibitors were investigated. The contents of 11 compounds were determined simultaneously for the comprehensive quality control of the* P. multiflorum* extract. The chromatogram of quantitative analysis is illustrated in Figure 3. After the* P. multiflorum* was processed, the contents of phenolic acid (gallic acid) and flavonoids (catechin and epicatechin) were enhanced, while the contents of stilbenes (polydatin, 2,3,5,4′-tetrahydroxystilbene-2-O-*β*-D-glucoside, and resveratrol) were reduced. Among the anthraquinones, the contents of physcion, rhein, and emodin were enhanced and those of physcion-8-O-glucoside and emodin-8-O-glucoside were depressed. The* P. multiflorum* extracts of different processing times were also calculated. The results were shown in Table 5. As can be seen from Table 5, the contents of gallic acid, catechin, and epicatechin had no regular changes with different processing times, while the contents of polydatin, 2,3,5,4′-tetrahydroxystilbene-2-O-*β*-D-glucoside, and resveratrol were decreased. The reason was that polydatin, 2,3,5,4′-tetrahydroxystilbene-2-O-*β*-D-glucoside, and resveratrol lose glycosides in the processing procedure. The contents of physcion, rhein, and emodin improved with the processing times until 32 h, after which the contents showed no regular changes. The concentrations of physcion-8-O-glucoside and emodin-8-O-glucoside presented a completely opposite tendency. The reason was that physcion-8-O-glucoside and emodin-8-O-glucoside were physcion and emodin derivative, respectively. Before 32 h, physcion-8-O-glucoside and emodin-8-O-glucoside were decreased while physcion and emodin were increased. But physcion and emodin were decreased after 32 h which might be related to other chemical reactions occurring in the process. The reason for this needs to be studied in further work. As a result, processing could affect the concentrations of bioactive components.

In order to understand the effect of processing on* P. multiflorum* more clearly, principal component analysis (PCA) was employed for exploring the effects of processing on quality of* P. multiflorum*. The sum of PC1 and PC2 was higher than 85.33% of total variance, which could be sufficient to describe the variability. The score plot for these compounds generated from comparison of two PCs is illustrated in Figure 3. 13 samples of different processing times were classified into 4 groups.* P. multiflorum* with no processing and 4 h processing were clustered into one group, respectively. Samples that were processed 8 to 16 h were classified into one group and 20 to 32 h were clustered into another group. Based on this, the quality control of* P. multiflorum* becomes more comprehensive; this could be employed as a standard for the herbal medicines in the market.

## 4. Conclusion

In this study, a bioactivity-based UHPLC-Q-TOF-MS-FC method was developed and validated to successfully screen and identify lipase inhibitors from* P. multiflorum*. The active components were used in clarifying the effects of processing on the quality of* P. multiflorum*. The method could provide the total activity of the extract, the activity of the factions and individual components, and the contribution of the active components in the herbal medicines. Compared with conventional methods, the developed method was more rapid, effective, and comprehensive for the active components screening and identification of lipase inhibitors in TCMs. As a result, stilbenes and anthraquinones in* P. multiflorum* were screened to have the potential lipase inhibitory effect, which could be used as safe lipase inhibitors for the treatment of obesity. These results give scientific support for the clinical use and specification for processing in the market for* P. multiflorum*.

## Figures and Tables

**Table 1:** UHPLC data for the calibration curves, LODs, LOQs, and repeatability (*n* = 6).

Compounds	Regressive equation	Linear range (*μ*g/mL)	*R* ^2^	LOD (*μ*g/mL)	LOQ (*μ*g/mL)	Repeatability RSD (%)
Gallic acid	*Y* = 5719*x* + 446.94	0.46~100	0.9997	0.15	0.46	2.14
Catechin	*Y* = 507.37*x* + 341.84	5.56~100	0.9994	1.85	5.56	1.59
Epicatechin	*Y* = 625.05*x* + 370.25	2.78~100	0.9977	0.93	2.78	3.97
Polydatin	*Y* = 1279.9*x* − 13.406	1.67~100	0.9975	0.56	1.67	2.35
2,3,5,4′-Tetrahydroxystilbene-2-O-*β*-D-glucoside	*Y* = 1271.8*x* + 1703.5	2.29~1000	0.9998	0.71	2.29	1.10
resveratrol	*Y* = 3466.9*x* − 263.63	0.56~100	0.9999	0.19	0.56	1.90
Emodin-8-O-glucoside	*Y* = 1518.4*x* + 174.91	1.11~100	0.9998	0.37	1.11	1.31
Physcion-8-O-glucoside	*Y* = 3563*x* + 1665.9	0.33~100	0.9955	0.11	0.33	1.69
Rhein	*Y* = 7705*x* − 1401	0.22~100	0.9996	0.07	0.22	1.70
Emodin	*Y* = 11056*x* − 743.8	0.19~100	0.9998	0.06	0.19	0.77
Physcion	*Y* = 2238.3*x* − 601.47	0.93~100	0.9996	0.31	0.93	1.92

**Table 2:** Intraday and interday precision and stability of the 11 compounds (*n* = 6).

Compounds	Concentration (*μ*g/mL)	Intraday		Interday		Stability	
		RSD (%)	Accuracy (%)	RSD (%)	Accuracy (%)	RSD (%)	Accuracy (%)
Gallic acid	1	4.31	96.6	1.35	97.6	4.77	106
	10	2.46	102	0.82	107	3.96	99.3
	100	2.57	97.2	0.75	99.3	3.49	97.3

Catechin	1	3.35	96.7	2.65	95	4.69	100
	10	1.98	100	1.94	101	4.98	95
	100	2.17	98.6	2.05	95.2	4.96	95.2

Epicatechin	1	1.85	95.2	1.44	94.7	2.26	96.7
	10	4.09	103	0.08	104	3.88	95.2
	100	3.79	94.7	0.15	94.2	4.23	103

Polydatin	1	0.90	98.2	2.17	95.3	4.70	98.2
	10	3.02	106	2.80	106	3.83	95
	100	3.12	93.8	2.75	98.2	3.96	106

2,3,5,4′-Tetrahydroxystilbene-2-O-*β*-D-glucoside	1	4.84	95	1.79	97.3	3.25	93.7
	10	3.50	102	0.45	100	4.87	96.4
	100	3.21	96.6	1.88	96.7	4.64	99.3

Resveratrol	1	4.32	102	2.15	102	3.94	97.1
	10	3.28	97.3	4.91	96.1	4.79	97.1
	100	3.29	99.3	0.81	93.7	2.91	99.5

Emodin-8-O-glucoside	1	2.81	101	1.80	103	4.16	96.1
	10	3.51	96.7	1.40	96.4	3.45	106
	100	3.76	95	0.98	96.4	3.24	93.7

Physcion-8-O-glucoside	1	5.00	99.8	2.12	97.1	2.76	96.4
	10	4.50	96.2	1.50	95.8	4.78	99.3
	100	4.68	95.2	1.45	95	4.97	97.1

Rhein	1	4.43	96.9	2.06	102	2.80	95
	10	4.20	95.8	2.80	93.7	3.86	102
	100	4.67	94.7	2.79	103	2.76	96.1

Emodin	1	4.69	104	2.76	96.4	4.91	96.3
	10	4.46	95	4.25	97.1	2.77	96.4
	100	4.28	94.2	3.57	95.8	3.04	97.3

Physcion	1	3.75	106	3.01	95	4.34	99.5
	10	4.95	96.4	2.23	102	3.77	97.2
	100	4.90	97.3	2.23	103	3.27	95.6

**Table 3:** The recovery of eleven bioactive compounds (*n* = 3).

Analyte	Original (*μ*g)	Spiked (*μ*g)	Found (*μ*g)	Average recovery (%)	RSD (%)
Gallic acid	3.52	2.82	6.21	95.3	0.88
		3.52	6.91	96.2	1.44
		4.23	7.90	104	1.85

Catechin	2.39	1.91	4.31	100	4.49
		2.39	4.85	103	0.61
		2.87	5.23	98.7	2.70

Epicatechin	9.08	7.26	16.4	101	0.73
		9.08	18.1	99.3	0.09
		10.9	20.3	103	0.33

Polydatin	15.8	12.7	28.3	98.6	3.93
		15.8	32.5	105	0.34
		19.0	35.4	103	0.83

2,3,5,4′-Tetrahydroxystilbene-2-O-*β*-D-glucoside	519	415	949	104	1.64
		519	1030	98.4	0.37
		623	1168	104	0.64

Resveratrol	9.14	7.31	16.7	103	1.06
		9.14	18.3	100	0.95
		11.0	19.9	98.4	1.59

Emodin-8-O-glucoside	21.3	17.1	39.1	104	1.86
		21.3	41.9	96.4	0.81
		25.6	47.1	101	2.87

Physcion-8-O-glucoside	7.86	6.29	14.4	104	1.57
		7.86	16.1	105	0.67
		9.43	17.6	104	0.82

Rhein	3.53	2.82	6.42	103	1.65
		3.53	6.95	97.2	1.29
		4.23	7.70	98.5	1.45

Emodin	5.79	4.63	10.5	102	0.77
		5.79	11.7	102	0.98
		6.95	13.0	104	1.06

Physcion	3.39	2.71	6.21	104	1.47
		3.39	6.64	95.9	1.07
		4.07	7.46	99.9	0.39

**Table 4:** UHPLC/Q-TOF-MS identification of lipase inhibitors and IC_50_ values.

Number	Rt (min)	MS	MS/MS	ppm	Formula	Compound	IC_50_ (*μ*g/mL)
1	1.409	169.0141		4.6	C_7_H_6_O_5_	Gallic acid	—
2	3.725	289.0724	215.0714, 173.0535, 149.0206, 125.0215, 109.0283	2.2	C_15_H_14_O_6_	Catechin	—
3	4.48	289.0724	276.4677, 205.0499, 163.0368, 131.0053, 109.0317	2.2	C_15_H_14_O_6_	Epicatechin	—
4	6.484	389.1168	227.1165	3.89	C_20_H_22_O_8_	Polydatin	1318
5	6.766	405.1184	243.0656, 137.0237	0.95	C_20_H_22_O_9_	2,3,5,4′-Tetrahydroxystilbene-2-O-*β*-D-glucoside	350.5
6	9.203	227.2002	202.0698, 176.0559, 99.9223, 91.0179, 73.5614	3.35	C_14_H_12_O_3_	Resveratrol	564.3
7	10.597	431.1386	269.0448, 225.0540	0.86	C_21_H_20_O_10_	Emodin-8-O-glucoside	296.0
8	11.836	445.0595	283.0595, 240.0415	0.76	C_22_H_22_O_10_	Physcion-8-O-glucoside	421.5
9	12.127	283.0606	240.0409	3.76	C_15_H_8_O_6_	Rhein	231.0
10	17.188	269.0460	241.0491, 225.0549, 210.0312, 197.0598	3.89	C_15_H_10_O_5_	Emodin	349.7
11	18.786	283.0610	240.0419, 116.9278	0.88	C_16_H_12_O_5_	Physcion	812.8

**Table 5:** Contents of 11 compounds in samples of different processed times (mg/g, *n* = 3).

Compounds	Contents (mg/g)												
	0 h	4 h	8 h	12 h	16 h	20 h	24 h	28 h	32 h	36 h	40 h	44 h	48 h
Gallic acid	0.35	0.84	1.02	0.58	1.09	1.10	1.45	1.25	1.84	2.45	1.69	1.97	1.73
Catechin	0.25	1.57	0.94	0.23	0.13	0.00	0.30	0.00	0.25	0.59	0.75	0.21	0.72
Epicatechin	1.05	0.94	1.36	0.42	0.53	0.26	0.45	0.45	0.60	0.60	0.43	0.39	0.48
Polydatin	1.57	1.34	1.04	0.87	0.77	0.73	0.68	0.59	0.55	0.48	0.42	0.33	0.32
2,3,5,4′-Tetrahydroxystilbene-2-O-*β*-D-glucoside	52.1	44.3	43.8	42.4	34.4	32.6	32.2	27.6	25.8	24.9	21.3	13.6	12.1
Resveratrol	0.99	0.97	0.77	0.73	0.69	0.29	0.61	0.39	0.54	0.80	0.52	0.39	0.37
Emodin-8-O-glucoside	5.40	4.05	2.62	2.38	2.16	2.22	1.96	1.63	1.41	1.31	0.98	0.79	0.68
Physcion-8-O-glucoside	1.79	1.47	1.18	1.12	1.09	1.12	1.09	0.80	0.67	0.52	0.30	0.04	0.03
Rhein	0.00	0.07	0.09	0.10	0.17	0.30	0.39	0.47	0.10	0.30	0.09	0.17	0.06
Emodin	0.58	1.46	2.08	3.08	3.20	3.39	3.64	3.76	2.31	3.11	1.47	2.62	1.34
Physcion	0.34	1.11	1.49	1.69	2.36	2.99	3.62	3.71	1.12	1.45	0.63	1.11	0.56
